# Larval abundances of rockfishes that were historically targeted by fishing increased over 16 years in association with a large marine protected area

**DOI:** 10.1098/rsos.170639

**Published:** 2017-09-20

**Authors:** Andrew R. Thompson, Dustin C. Chen, Lian W. Guo, John R. Hyde, William Watson

**Affiliations:** 1 NOAA Fisheries Service, Southwest Fisheries Science Center, 8901 La Jolla Shores Drive, La Jolla, CA 92037-1508, USA; 2 Department of Environmental and Ocean Sciences, University of San Diego, San Diego, CA 92110, USA; 3 Organismic and Evolutionary Biology, University of Massachusetts Amherst, 611 N. Pleasant Street, Amherst, MA 01003-9297, USA

**Keywords:** fisheries management, marine protected area, rockfish, California, marine conservation

## Abstract

Marine protected areas (MPAs) can facilitate recovery of diminished stocks by protecting reproductive adults. To effectively augment fisheries, however, reproductive output must increase within the bounds of MPAs so that larvae can be exported to surrounding areas and seed the region. In response to dramatic declines of rockfishes (*Sebastes* spp.) in southern California by the late 1990s two large MPAs, the Cowcod Conservation Areas (CCAs), were established in 2001. To evaluate whether the CCAs affected rockfish productivity we evaluated the dynamics of 8 species that were, and 7 that were not, historically targeted by fishing. Abundances of 6/8 targeted and 4/7 non-targeted species increased regionally from 1998 to 2013. These upturns were probably affected by environmental conditions in addition to changes in fishing pressure as the presence of most species correlated negatively with temperature, and temperature was lower than the historic average in 11/15 years. Seventy-five per cent of the targeted, but none of the non-targeted species increased at a greater rate inside than outside the CCAs while controlling for environmental factors. Results indicate that management actions, coupled with favourable environmental conditions, facilitated the resurgence of multiple rockfish species that were targeted by intense fishing effort for decades.

## 1. Introduction

Negative impacts of fishing on populations and ecosystems have been known for decades [1,2]. The problem continues to this day, as significant depletions and declining catch-per-unit-effort (CPUE) were found to be a recurring trend worldwide [3]. Further, predatory fish biomass is estimated to have declined substantially across the globe from 1880 to 2007, and the rate of decline of top predators is still accelerating while prey abundances are increasing, probably due to predator-release effects [4]. Determining how to alleviate overfishing, therefore, is one of the most important issues for both fisheries science and management.

Marine protected areas (MPAs) are ecological refuges that hold the potential to assuage the effects of overfishing, especially for relatively sedentary species, and have been implemented globally over the past two decades [5,6]. Although it has been shown worldwide that animal abundance, size and species richness often increases within MPA bounds [5,7,8], MPA effects must transcend the local MPA area to benefit fisheries [9,10]. Movement of adults beyond MPAs (i.e. spillover) is one mechanism by which MPAs can augment fisheries [11], but this effect seems to mostly be limited to areas adjacent to MPA boundaries [12]. The main way that MPAs can improve fisheries at a large scale is by increasing larval production; larvae can then be transported from the MPA and provide a ‘recruitment subsidy’ [13] to surrounding fished regions [14]. Despite the importance of larval production to MPA efficacy we are not aware of research that has quantitatively assessed the effects of an MPA on long-term reproductive output. In this study we evaluate rockfish (*Sebastes* spp.) larval production throughout southern California at both regional and MPA scales.

One difficulty in evaluating MPA effects has been a lack of robust sampling designs [15,16]. MPA impacts may be masked or misinterpreted if, for example, samples are collected only within MPA bounds without outside control locations. Furthermore, inside and outside locations should be paired such that habitat conditions are similar inside and outside of MPAs [5]. An ideal set-up will monitor both before and after MPA establishment to determine if sample (e.g. fish abundances) trajectories diverge inside and outside following placement of MPAs [15]. Finally, data should be collected on species that are and are not targeted by fishing to assess if MPAs are affecting population dynamics of protected species [17]. We use a before--after, control-impact paired series design [18] to quantify MPA effects on larval abundances of 15 rockfish species that were and were not historically targeted by fishing.

Rockfishes (*Sebastes* spp.), are a speciose (102 described species as of 2002 [19]) group of demersal fishes found mostly (96 species [19]) along the west coast of North America and the Gulf of California. Whereas some species are relatively small and short-lived forage fish, others grow to large sizes (more than 90 cm), live for many decades (more than 150 years), and are apex predators [19]. Larger rockfish species have been targeted by fisheries since at least the mid-nineteenth century, and technological developments subsequent to the 1940s, and in the 1970s in particular, led to severe population declines due to overfishing by the late 1990s [19]. A survey of commercial passenger fishing vessels in the Southern California Bight (SCB) region from 1980 to 1996 revealed drastic declines in CPUE and mean total length of multiple rockfish species such as bocaccio (*S. paucispinis*), blue (*S. mystinus*), olive (*S. serranoides*), chilipepper (*S. goodei*), swordspine (*S. ensifer*), yellowtail (*S. flavidus*) and vermilion rockfishes (*S. miniatus*) [20]. Fishery-independent surveys of larval fishes from 1977 to 1998 also documented declines in abundances of bocaccio and cowcod (*S. levis*) that were attributed to changing environmental conditions (a shift from cool to warm climate regime in the late 1970s) and fishery exploitation [21].

**Figure 1.**
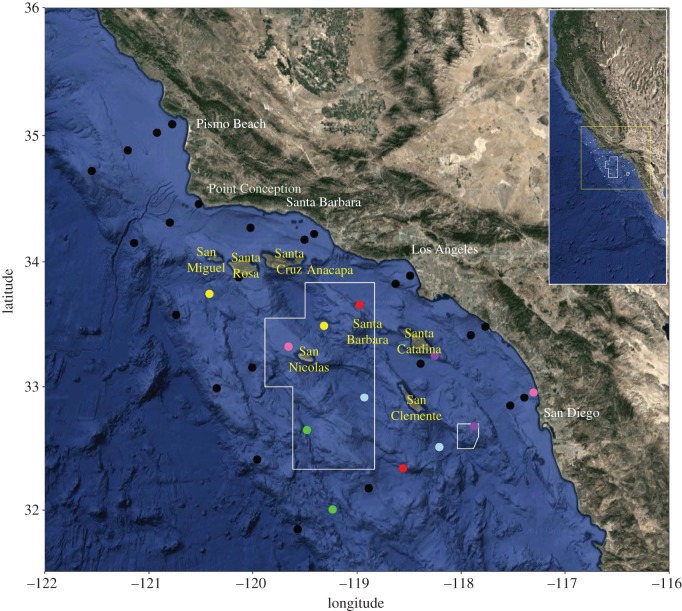
Location of stations. Colours designate stations with similar environmental conditions. These stations were used to determine whether rates of change in larval abundance differed inside and outside of the Cowcod Conservation Areas. Boundaries of the fine-scale map are shown in yellow on the large-scale inset map.

In response to the decline in populations of rockfishes in southern California, (particularly cowcod, which was formally declared overfished in 1999 [22]), the Pacific Fishery Management Council established two Cowcod Conservation Areas (CCAs) in 2001. The CCAs comprise a western and eastern area in the SCB (encompassing 10 878 km^2^ and 260 km^2^, respectively; figure 1) where cowcod were historically caught at high rates [22]. Bottom-fishing deeper than 36 m is prohibited within the CCAs as larger, targeted rockfishes typically reside below this depth. These areas are several times larger than most marine MPAs that have been comprehensively studied thus far (but see [23]) and are the largest rockfish conservation areas in the world.

Previous research suggests that the CCAs are benefitting targeted rockfishes [24–26]. However, there have been no studies assessing how the CCAs have impacted rockfishes through time. Both stock assessments [27,28] and ecological studies [29,30] conducted in and around the CCAs noted that the lack of time-series data hinders our capacity to understand whether the CCAs have influenced the rebuilding of rockfish stocks. We analyse a unique fishery-independent time series of rockfish larvae collected annually from 1998 to 2013 within and outside of the MPAs, before and after MPA establishment. The dataset includes species that are both targeted and untargeted by fishers, as well as oceanographic and habitat conditions at systematically sampled locations. We are thus able to evaluate MPA effects while controlling for oceanographic dynamics and fishing effort. We ask three primary questions: (i) Has there been a systemic change in rockfish production during the 16-year study period throughout southern California? (ii) Does environmental variability explain changes in larval abundances? (iii) Are population trajectories different within versus outside of the CCAs when controlling for environmental effects?

## 2. Methods

### 2.1. Sample collection

The California Cooperative Oceanic Fisheries Investigations (CalCOFI) programme has been regularly monitoring fixed stations in the SCB since 1949 [31]. CalCOFI uses 0.71 m diameter, 505 µm-mesh bongo nets towed obliquely from a depth of 210 m to collect plankton samples, and conductivity, temperature, and depth instruments (CTDs) to record oceanographic variables (e.g. temperature, salinity, dissolved oxygen concentration, and chlorophyll *a*) [31]. From 1949 until 1997 samples were fixed and stored in 5% formalin but subsequent to 1997 samples from the portside bongo net were preserved in 95% ethanol (formalin degrades while ethanol preserves DNA; contents of the starboard net continued to be placed in formalin). This study focuses on ethanol-preserved samples collected annually from 1998 to 2013 during the winter (January--February; the peak spawning period for the majority of rockfishes in this region) from 36 CalCOFI stations (figure 1) located over the continental shelf [32,33]. Serendipitously, 6 CalCOFI stations were within the CCAs, and ethanol preservation began before the CCAs were established in 2001.

### 2.2. Species identification and data refinement

Ichthyoplankton that were visually identified as rockfishes were removed, counted, and measured for total length with a dissecting microscope and micrometer. A few rockfish species, such as shortbelly, bocaccio, and older stages of cowcod can be morphologically identified to species, and these were enumerated visually under a microscope. The vast majority of rockfish larvae, however, are not identifiable to species based on morphology, and these were identified genetically by sequencing the mitochondrial cytochrome *b* gene (see the electronic supplementary material, appendix S1) and matching sequences to a genetic library developed by Hyde & Vetter [34]. Previous analyses demonstrated that this gene can discriminate all rockfish species from southern California [26,35]. All larvae that were morphologically identified as cowcod and bocaccio were sequenced to confirm their identity, but most shortbelly were not sequenced as previous analyses indicated that 100% of visually identified shortbelly were indeed this species [26]. Unfortunately, most larvae from 2003 were poorly preserved and unable to be sequenced; we thus excluded 2003 samples from the analyses. We also eliminated from analyses stations where at least 50% of the larvae failed to sequence. These eliminated stations were randomly distributed and thus probably did not induce any systematic bias to the analyses. To standardize for minor differences in tow lengths and/or depths among stations, larval count data were multiplied by a standard haul factor (SHF), which is calculated by dividing the volume of water filtered by the tow depth and expressed as larvae under 10 m^2^ of sea surface area to the depth of a tow (200 m or 10 m from the bottom for shallow stations) [36,37].

### 2.3. Regional abundance dynamics

We tested whether larval abundance systematically changed throughout southern California by correlating mean larval abundance per year against year with a general linear model. Annual winter means were calculated using the delta-mean technique that helps account for high numbers of samples containing zero values [38]. Analyses were restricted to common species (at least 300 larvae under 10 m^2^ summed across all years). We also removed an extreme outlier station in 2004 that contained abundances of cowcod, pygmy and bocaccio that were between approximately two and three times greater than the next highest station in the entire dataset (electronic supplementary material, figure S1). We calculated the proportion of species that were historically targeted and non-targeted by fishing (as defined by Love *et al*. [19]) that increased significantly (*p* < 0.10) through time. Our goal was to determine if there were similar patterns between targeted and untargeted rockfishes rather than determine the significance for any one species; therefore, we did not apply a Bonferroni correction.

### 2.4. Environmental influence

In addition to cessation of fishing pressure, environmental conditions can contribute to changes in larval rockfish abundances. To determine dynamic habitat preference for the common species, we used generalized linear models (family = binomial, link = logit) to test whether the presence/absence (we initially used two-stage models that evaluated environmental effects separately on presence/absence and abundances of non-zero stations but found that the abundance component did not improve model performance) of larvae at each station and year was affected by temperature (°C), salinity (psu), oxygen (ml l^−1^) and chlorophyll *a* (μg l^−1^). Covariance was low among these independent variables (*r* < 0.57) and all were included in the analyses. Because the vast majority of rockfish larvae occupy the upper 100 m of the water column [39], we computed mean values for each environmental covariate between 3 and 100 m. We limited these analyses to include only larvae that were less than or equal to 5 mm total length (TL) based on previous findings that these young larvae had mostly not been advected far from their natal location [24,26]. Residuals for each species model were not found to be spatially autocorrelated but were temporally autocorrelated (electronic supplementary material, figure S2). To account for temporal autocorrelation we included year as an autocovariate; residuals of these models were not temporally autocorrelated (electronic supplementary material, figure S3). We again calculated the proportion of targeted and untargeted species whose presence/absence correlated significantly (*p* < 0.10) with a covariate. To provide a sense of how oceanographic conditions during the study compared with long-term patterns, we calculated yearly winter averages of the environmental variables from the time-series data and compared them to long-term winter averages obtained from CalCOFI hydrographic data from 1983 to 2013.

### 2.5. Cowcod Conservation Area influence

If the CCAs positively influenced rockfish production, we would expect larval abundances to increase at a greater rate inside compared to outside of the protected areas for targeted species, but not for untargeted species. However, environmental conditions can also affect production dynamics and thus obscure MPA effects. To isolate CCA from environmental effects, we paired each of the six stations within the CCA to one outside of the MPA. Pairs were chosen based on habitat similarity. We conducted a Bray–Curtis cluster analysis on stations based on means from 1998 to 2013 of the four environmental variables (oxygen, chlorophyll *a*, temperature and salinity), as well as two stationary variables, depth and percentage of hard substrate, that are known to affect adult rockfish distribution [26,32]. Proportion of hard substrate was obtained from the Seafloor Mapping Lab at California State University, Monterey Bay (http://seafloor.otterlabs.org/contact.html) while depth was measured with shipboard instruments. We then selected six stations outside of the protected areas that most closely matched habitat conditions of the six CCA stations (electronic supplementary material, figure S4). Next, we calculated annual delta means for each species with a total abundance of ≥150 larvae under 10 m^2^ during the study for the six stations inside and six outside of the CCAs. Four targeted species, bank*,* bocaccio*,* speckled and olive, and six non-targeted species, squarespot, shortbelly, pygmy, stripetail, swordspine and whitespeckled, were abundant enough to be analysed. Blue rockfish also met the abundance threshold but was excluded because it was almost always found north of the Channel Islands and thus was unable to be affected by the CCAs (electronic supplementary material, figure S5). As with the previous analysis we only used individuals that were less than or equal to 5 mm TL. We then conducted a type III analysis of covariance (ANCOVA) for each species with year, station location (inside/outside the CCAs) and an interaction between these terms as the dependent variables. This interaction was particularly important as significance indicates that abundances changed at a different rate inside versus outside the CCA. We thus determined the proportion of targeted and untargeted species where the *p*-value for the interaction between year and CCA was less than 0.10. All analyses and figures were made using the statistical analysis software R v. 3.2.3 [40]. R packages are described in electronic supplementary material, appendix S2.

## 3. Results

### 3.1. Overview

We processed 6717 larvae and identified 39 rockfish species throughout the time-series (electronic supplementary material, table S1). Two non-targeted species, squarespot and shortbelly, were especially common, comprising over 50% of the combined larvae (table 1). The next most abundant non-targeted species were pygmy, halfbanded, stripetail, swordspine and whitespeckled (table 1). The most abundant targeted species were bocaccio, blue, bank, speckled, olive, widow, chilipepper and copper (table 1). Conversely, some species were extremely rare. For example, we detected only one individual (not adjusting for SHF) for greenspotted (*S. chlorostictus*), greenblotched (*S. rosenblatti*) and flag (*S. rubrivinctus*), two individuals for calico (*S. dalli*) and yelloweye (*S. ruberrimus*), and three for Mexican (*S. macdonaldi*) (electronic supplementary material, table S1).

**Table 1.** Abundances of the 15 analysed species summed over the 16-year study period. Abundance is expressed in no. larvae under 10 m^2^ (no. larvae multiplied by a standard haul factor) and number of larvae is the raw number for each species. Fishing pressure is based on descriptions by Love *et al*. [19]. A complete list of genetically identified species is shown in electronic supplementary material, table S1.

species	common name	fishing pressure	abundance	no. larvae
*S. hopkinsi*	squarespot	low	9430	2171
*S. jordani*	shortbelly	low	6965	1494
*S. wilsoni*	pygmy	none	2254	489
*S. paucispinis*	bocaccio	high	1508	330
*S. mystinus*	blue	high	1508	328
*S. semicinctus*	halfbanded	low	1371	323
*S. rufus*	bank	high	1053	228
*S. saxicola*	stripetail	low	1047	226
*S. ovalis*	speckled	high	808	183
*S. goodei*	chilipepper	high	717	143
*S. ensifer*	swordspine	low	557	119
*S. serranoides*	olive	moderate	461	106
*S. moseri*	whitespeckled	none	395	86
*S. entomelas*	widow	low	344	72
*S. caurinus*	copper	high	324	72
*S. levis*	cowcod	high	195	43

### 3.2. Temporal trends

**Figure 2.**
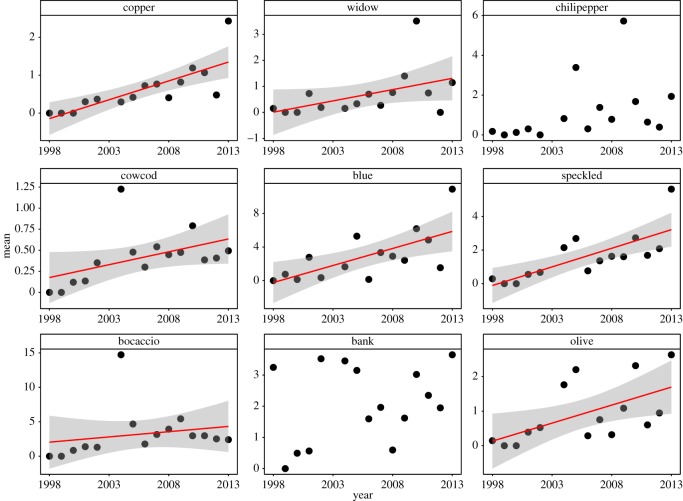
Change in mean larval abundances through time for species targeted by fishing. Red line (best linear fit) and shading (95% confidence interval) are shown for species where there was a significant relationship between mean abundance and year.

**Figure 3.**
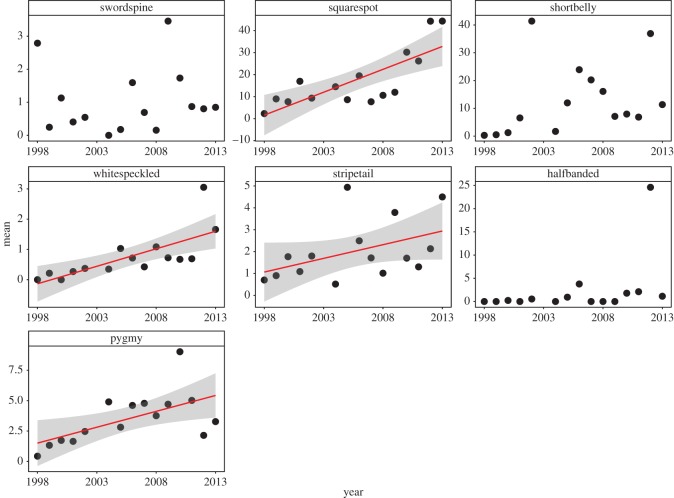
Change in mean larval abundances through time for species not targeted by fishing. Red line (best linear fit) and shading (95% confidence interval) are shown for species where there was a significant relationship between mean abundance and year.

Six of the eight (excluding cowcod which were quite rare) most abundant targeted species (copper, widow, blue, speckled, bocaccio and olive) showed significant increases in their mean abundances across the entire region over time (figure 2; electronic supplementary material, table S2). Interestingly, although cowcod abundances were below the threshold for formal analysis, this species did significantly increase during the study (figure 2). Four of the seven (squarespot, whitespeckled and pygmy) most abundant non-targeted species significantly or nearly significantly (stripetail) increased during the study (figure 3; electronic supplementary material, table S2).

### 3.3. Environmental influence

The probability of presence of six of the eight most abundant targeted species (copper, widow, blue, speckled, bocaccio and olive) correlated negatively with temperature (table 2; electronic supplementary material, table S3*a*). Two of the seven most abundant non-targeted species (whitespeckled and pygmy) also had negative relationships with temperature (table 2; electronic supplementary material, table S3*b*). There were also significant positive relationships with chlorophyll *a* for copper, squarespot, shortbelly, whitespeckled and halfbanded (table 2; electronic supplementary material, table S3). Trend direction with salinity and oxygen were inconsistent as the presence of chilipepper decreased but swordspine increased with salinity (table 2; electronic supplementary material, table S3). Similarly, trends were positive with oxygen for blue but negative for shortbelly and halfbanded (table 2; electronic supplementary material, table S3).

**Table 2.** Slopes of logistic regression coefficients for targeted (upper) and untargeted rockfishes. A * denotes significance at *p* < 0.10, **p < 0.05, ***p < 0.01, ****p < 0.001

coefficient	copper	widow	chilipepper	blue	speckled	bocaccio	bank	olive
temperature	−0.55	−0.9***	−0.20	−1.080****	−0.44*	−0.74****	−0.12	−0.63**
salinity	0.88	−1.32	−3.21**	−0.38	−0.64	−1.47	0.25	−1.14
oxygen	0.24	0.51	−0.90	1.021**	0.00	0.31	0.54	0.24
chlorophyll *a*	0.49**	0.00	−0.02	0.25	0.09	−0.04	−0.20	0.14
year	0.098*	0.03	0.12**	0.080**	0.12***	0.059*	0.081***	0.07

coefficient	swordspine	squarespot	shortbelly	whitespeckled	stripetail	halfbanded	pygmy	
temperature	0.01	−0.16	0.01	−0.68**	−0.10	0.22	−0.74****	
salinity	3.14**	0.04	−1.26	−0.28	−0.99	−2.24	−0.87	
oxygen	0.35	−0.51	−1.27****	0.92	−0.63	−1.31**	0.10	
chlorophyll *a*	−0.41	0.29*	0.49***	−1.45**	0.28	0.61***	−0.03	
year	0.05	0.070***	0.070**	0.12***	0.04	0.25****	0.03	

**Figure 4.**
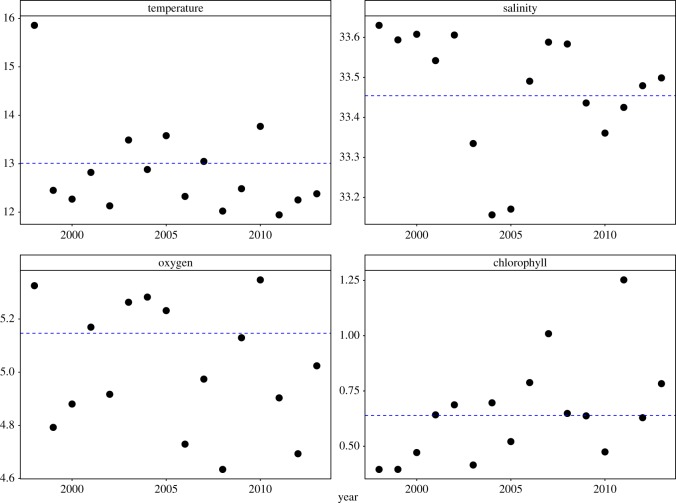
Temperature (°C), salinity (psu), oxygen (ml l^−1^) and chlorophyll *a* (μg l^−1^) in the upper 3–100 m averaged across all stations for each year. Blue lines are annual winter averages between 1982 and 2013 from the same stations.

Average winter values of the environmental variables during the study period (1998–2013) compared to long-term averages (1983–2013) indicated that temperature was frequently lower (11 out of 16 years), salinity was frequently higher (10 out of 16 years), and oxygen was frequently lower (10 out of 16 years) during the study (figure 4). Chlorophyll *a* levels fluctuated nearly equally below and above the long-term winter average (seven years above, eight below, and one value nearly identical to the long-term average) (figure 4).

### 3.4. Cowcod Conservation Area influence

**Figure 5.**
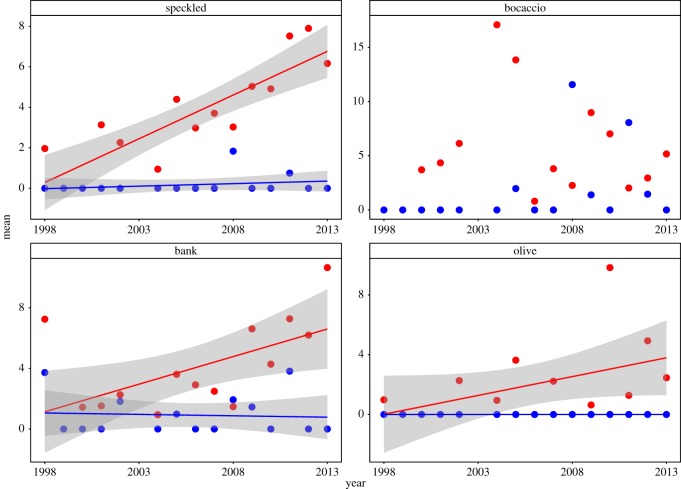
Relationship between larval abundance and year for targeted species with at least 150 larvae under 10 m^2^ summed across 12 paired stations (six inside and six outside of the CCAs) during the study. Red and blue colours depict stations within and outside of the CCAs, respectively. Best-fit linear regression lines and 95% confidence intervals are shown for species where there was a significant interaction between year and CCA.

The interactions between year and CCA were significant for three out of four of most abundant targeted species (speckled, bank, olive) (figure 5; electronic supplementary material, table S4*a*). The interaction was not significant (*p* between 0.17 and 0.99) for any of the seven most abundant untargeted species (electronic supplementary material, figure S4 and table S4*b*).

## 4. Discussion

We found that larval abundances of the majority of targeted rockfishes increased throughout southern California between 1998 and 2013. The rates of increase, however, were much higher within than outside of the CCAs for most of the targeted but none of the untargeted species (figure 5; electronic supplementary material, table S4). This indicates that the presence of the CCAs is facilitating the recovery of rockfish species that were historically targeted by fishers.

Although it is recognized that augmenting reproductive output is crucially important for MPA success [13,41], to our knowledge this is the first demonstration that an MPA affected larval production at such large spatial and temporal scales. A handful of studies, however, measured reproductive output in association with MPAs at smaller scales. A study in and around a small (0.9 km^2^) MPA in the NW Mediterranean in 2003 found high species richness and high abundance of larvae from targeted species in the MPA and suggested that larval export could seed the surrounding fished areas [42,43]. Another study in the Mediterranean in 2004 also found that commercially targeted fishes were spawning at relatively high rates within an MPA and that the resulting high abundance of larvae were being exported to outside, fished locations [44]. Similarly, larval abundance of commercially harvested queen conch, *Strombus gigas*, were an order of magnitude higher within than outside of a fished area in the Bahamas and probably seeded fished areas north of the MPA [45]. In addition, studies from South Africa [46], Spain [47] and New Zealand [48] indirectly estimated larval production based on female biomass and concluded that fecundity was higher within than outside of MPAs. Our results, in conjunction with these studies, suggest that MPAs often have higher rates of larval output than surrounding regions, with larval movement allowing for recovery of fish populations beyond the boundaries of an MPA.

While larval rockfish abundances (and larval fish abundances in general) can be driven by multiple factors, it is likely that increased biomass of reproductively active females and favourable environmental conditions contributed to the larval abundance dynamics observed in our study. We found that larval abundances of the majority of both targeted and non-targeted rockfishes increased throughout southern California between 1998 and 2013. Increasing adult rockfish abundances were also documented in stock assessments on *S. levis* in the SCB and *S. paucispinis* along the western US coast [27,28]. These trends were probably influenced by exceptionally high recruitment for most species in 1999 when there was a strong La Niña with cold and productive waters [49]. Submersible surveys [25] also support the idea of high recruitment in 1999 as cowcod were 51–60 cm TL in 2012 which, based on von Bertalanffy growth curves [50], places their birth date around 1999. Given that management limited fishing beginning in 2000, it is probable that a relatively large proportion of the 1999 cohort survived long enough to begin becoming reproductively active and contributed to larval production by 2004.

In addition to management actions that allowed more individuals to reach maturity, environmental conditions probably contributed to the proliferation of rockfish larvae. Rockfish spawning output is affected by the environment as female reproduction is reduced when food is scarce and adult energy reserves are low [19]. Low spawning years typically occur during El Niños when water temperature is high and primary productivity is low [51,52]. Our logistic regression models support the idea that reproduction is higher when the water is cool as the presence of most species correlated negatively with temperature. Further, we found that the water was cooler than the 30-year average in most years between 1998 and 2013, and it has been speculated that 1999 marked the beginning of an oceanographic shift from warm conditions that characterized the region between 1977 and 1998 [53]. Therefore, environmental conditions appeared to have been generally conducive for high larval production and recruitment throughout much of the study.

Whereas overfishing remains a global concern [54], synergistic effects of management and favourable ocean conditions have been identified to augment the recovery of another long-lived, overfished species [55]. Striped bass (*Morone saxatilis*) in the Chesapeake Bay, USA, were severely overfished throughout the 1970s and 1980s. In 1984 a strict fishing moratorium was implemented, and favourable environmental conditions produced strong recruitment in 1989, 1993 and 1996 [56,57]. By 2000, the stock was rebuilt to pre-exploitation levels [57]. Similarly, SCUBA surveys within and outside of no-take MPAs around the Channel Islands in California demonstrated that targeted reef fishes such as cabezon (*Scorpaenichthys marmoratus*) and kelp rockfish (*Sebastes atrovirens*) increased both within and outside of reserves between 2003 and 2012 [17]. It is possible that targeted rockfishes and other long-lived, targeted species throughout southern California have responded positively to management action and cool, productive ocean environments.

Although this was the first study to examine rockfish dynamics in association with the CCAs, short-term studies also suggest that the CCAs benefit rockfish populations. A larval survey in 2005 using the same techniques as the current work but with finer-scale sampling showed that species richness and the abundance of targeted rockfishes was higher within than outside of the CCAs [26]. Similarly, submersible surveys in 2012 encountered cowcod with greater frequency within than outside of the CCAs [25]. In addition, abundances of recently hatched, but not older, larval bocaccio were concentrated around the relatively shallow banks within the eastern CCA [24]. Although we did not detect an interaction between year and CCA for bocaccio, abundances were higher within than outside of paired CCA stations in all but two years. These studies and ours indicate that the CCAs were positioned in locations that are well suited to protect and facilitate the recovery of many rockfishes in southern California.

Relatively large Rockfish Conservation Areas (RCAs) have also been established along the western US coast, as well as further north in Canadian waters with the goal of facilitating recovery of overfished stocks. US RCAs north of southern California appear to be benefitting rockfishes in addition to other demersal species as both bottom trawl [58] and hook and line surveys [59] detected significant increases in abundances since the beginning of the millennium. However, studies in Canada obtained mixed results. Whereas surveys in the Strait of Georgia indicated that these RCAs positively impacted rockfishes [60], a more comprehensive study suggested that Canadian RCAs have not facilitated recovery of demersal fish populations [61]. Lack of compliance was a potential explanation for the Canadian MPA ineffectiveness as there was no difference in fishing activity before and after establishment in most of these RCAs [62]. Indeed, a recent global survey found that many MPAs were inadequately managed and these performed almost three times worse than equitably governed MPAs [63]. It is likely that differences in management efficacy explain performance variation between the US and Canadian RCAs.

Although larval abundances of several targeted rockfishes increased throughout the time-series, targeted larvae were far outnumbered by non-targeted species. Given that the ratio of abundances of a targeted (bocaccio) to non-targeted (shortbelly) species was significantly lower between 1976 and 2001 versus 1951 to 1975 [64], that shortbelly were much more common than bocaccio in this study, and that cowcod larval abundances remained low, it seems that targeted rockfish populations have not returned to pre-exploitation levels even within the CCAs. It is possible that not enough time has elapsed for management effects to be fully manifested in some targeted species populations. Theoretical models indicate that it may take decades for targeted rockfish species to reach carrying capacity following MPA establishment because these species experienced high fishing pressure, undergo maturation at older ages, and have low rates of natural mortality [65]. Empirical work in the Mediterranean also shows that large fishes that were heavily exploited can take multiple decades to reach equilibrium levels [66]. Alternatively, the system could be in an alternative stable state, having transitioned from being dominated by larger targeted rockfish species to smaller, faster-growing, non-targeted species [67]. If this is the case, the smaller species may be consuming young targeted species and hence directly impeding recovery. Further monitoring is needed to evaluate whether larval abundances of targeted rockfishes continue to increase relative to non-targeted species after 2013.

Increasing larval rockfish abundances in the CCAs hold the potential to positively affect rockfish populations at a regional scale through larval spillover [41]. Recent work off the Great Barrier Reef, Australia, and Hawaii demonstrated through genetic parentage analysis that targeted species that were born within a MPA settled and recruited outside of MPAs in locations open to fishing [68,69]. Although this type of analysis is beyond the scope of our data, the next step is to use oceanographic models to more precisely estimate the origin and trajectories of the rockfish larvae in our study. Depending on the species, rockfishes spend 1–2 months as pelagic larvae and then up to a year as pelagic juveniles [19]. As such, they have the potential to disperse beyond MPA boundaries. In the future we plan to age larvae by counting daily otolith rings and use regional oceanic modelling systems (ROMS) models [70] to trace the path each larvae took to arrive at its location of capture and where it probably would have gone had it not been captured. This work should help better establish the degree to which the CCAs are providing a recruitment supplement to fished areas.

Our research underscores the value of long-term marine monitoring programmes such as CalCOFI to fisheries management and conservation. Whereas the initial focus of CalCOFI was to determine why the Pacific sardine fishery collapsed in the early 1950s, it has evolved into a comprehensive ecosystem monitoring programme that tracks the dynamics of the physical ocean and hundreds of species of fishes and invertebrates [31]. The fact that CalCOFI stations were placed systematically throughout southern California allowed us to quantify MPA effects with samples collected before and after and within and outside of the MPAs. This type of sampling design is rare in MPA studies but is necessary to unambiguously discern MPA effects [15,71,72]. Our work emphasizes the need to maintain programmes such as CalCOFI to help understand how anthropogenic impacts and climate continue to interact and influence fisheries dynamics.

Our work strongly indicates that the CCAs have been effective in facilitating the recovery of multiple targeted rockfish species and supports the effectiveness of establishing and regularly monitoring long-term MPAs. Given that augmenting larval output is the primary mechanism by which MPAs can benefit fisheries, this study provides an example of how larval monitoring can be used to assess MPA efficacy.

## Supplementary Material

Raw larval abundance and environmental data for Thompson et al.

## Supplementary Material

Supplemental Tables for Thompson et al. 2017

## Supplementary Material

Supplemental Figures for Thompson et al. 2017

## Supplementary Material

Supplemental Appendices for Thompson et al. 2017

## Data accessibility

Data analysed are available as electronic supplementary material. In addition, all data are publicly accessible via the CalCOFI ERDDAP website (http://coastwatch.pfeg.noaa.gov/erddap/tabledap/index.html?page=1&itemsPerPage=1000).
