# Supplementary material for: Larval abundances of rockfishes that were historically targeted by fishing increased over 16 years in association with a large marine protected area
Source: R Soc Open Sci. 2017 Sep 20;4(9):170639. doi: 10.1098/rsos.170639 (PMC5627106; doi:10.1098/rsos.170639)
Supplement: Supplemental Tables for Thompson et al. 2017 [file rsos170639supp2.pdf]

Supplementary Tables

Thompson et al. 2017 Production of rockfish historically targeted by fishing has increased in a large marine protected area over 16 years. Proc B

Table S1. Complete list of all rockfish larvae that were identified.

species	common name	fishing pressure	abundance (no. under 10 m^2)	no. larvae
S. hopkinsi	square spot	low	9430	2171
S. jordani	shortbelly	low	6965	1494
S. wilsoni	pygmy	none	2254	489
S. paucispinis	bocaccio	high	1508	330
S. mystinus	blue	high	1508	328
S. semicinctus	halfbanded	low	1371	323
S. rufus	bank	high	1053	228
S. saxicola	stripetail	low	1047	226
S. ovalis	speckled	high	808	183
S. goodei	chillipepper	high	717	143
S. ensifer	swordspine	low	557	119
S. serranoides	olive	moderate	461	106
S. moseri	whitespeckled	none	395	86
S. entomelas	widow	low	344	72
S. caurinus	copper	high	324	72
S. diploproa	splitnose	moderate	254	55
S. crocotulus	sunset	high	241	51
S. levis	cowcod	high	195	43
S. rufinanus	dwarf-red	none	150	33
S. rosaceus	rosy	moderate	130	29
S. aurora	aurora	moderate	127	26
S. miniatus	vermilion	high	123	26
S. elongatus	greenstriped	moderate	117	25
S. melanostomus	black	high	53	11
S. flavidus	yellowtail	high	38	8
S. phillipsi	chameleon	low	33	7
S. rastrelliger	grass	high	19	4
S. helvomaculatus	rosethorn	moderate	19	4
S. gilli	bronzespotted	low	18	4
S. simulator	pinkrose	low	15	3
S. constellatus	starry	high	15	3
S. auriculatus	brown	moderate	13	3
S. dalli	calico	low	13	3
S. macdonaldi	mexican	low	13	3
S. ruberrimus	yelloweye	high	10	2
S. rosenblatti	greenblotched	moderate	5	1
S. crameri	darkblotched	high	5	1
S. rubrivinctus	flag	moderate	4	1
S. chlorostictus	greenspotted	high	4	1

Table S2. Complete ANOVA table for analyses of mean larval abundance ~ year for each analyzed species. Coefficients that are significant at $p < 0.10$ are in bold.

species	coefficient	estimate	se	t	p	model adj. r^2	model p
copper	**intercept**	**-198.75**	**44.26**	**-4.49**	**0.001**	**0.58**	**0.0006**
	**year**	**0.10**	**0.02**	**4.50**	**0.001**		
widow	**intercept**	**-174.79**	**90.71**	**-1.93**	**0.076**	**0.16**	**0.075**
	**year**	**0.09**	**0.05**	**1.93**	**0.075**		
chilipepper	intercept	-259.67	162.77	-1.60	0.14	0.10	0.13
	year	0.13	0.08	1.60	0.13		
cowcod	**intercept**	**-73.17**	**17.01**	**-4.30**	**0.001**	**0.56**	**0.0008**
	**year**	**0.04**	**0.01**	**4.32**	**0.001**		
blue	**intercept**	**-810.93**	**251.07**	**-3.23**	**0.01**	**0.40**	**0.006**
	**year**	**0.41**	**0.13**	**3.24**	**0.01**		
speckled	**intercept**	**-457.80**	**104.09**	**-4.40**	**0.001**	**0.57**	**0.001**
	**year**	**0.23**	**0.05**	**4.41**	**0.001**		
bocaccio	**intercept**	**-393.74**	**170.61**	**-2.31**	**0.038**	**0.24**	**0.037**
	**year**	**0.20**	**0.09**	**2.32**	**0.037**		
bank	intercept	-157.60	128.00	-1.23	0.24	0.038	0.24
	year	0.08	0.06	1.25	0.24		
olive	**intercept**	**-221.13**	**75.90**	**-2.91**	**0.01**	**0.35**	**0.01**
	**year**	**0.11**	**0.04**	**2.93**	**0.01**		
swordspine	intercept	-19.35	113.17	-0.17	0.87	-0.07	0.86
	year	0.01	0.06	0.18	0.86		
square spot	**intercept**	**-4147.11**	**945.57**	**-4.39**	**0.001**	**0.57**	**0.0007**
	**year**	**2.08**	**0.47**	**4.40**	**0.001**		
shortbelly	intercept	-1661.95	1380.90	-1.20	0.25	0.25	0.25
	year	0.84	0.69	1.21	0.25		
whitespeckled	**intercept**	**-231.16**	**60.39**	**-3.83**	**0.002**	**0.50**	**0.002**
	**year**	**0.12**	**0.03**	**3.84**	**0.002**		
stripetail	intercept	-248.63	138.76	-1.79	0.10	0.14	0.09
	**year**	**0.12**	**0.07**	**1.81**	**0.09**		
halfbanded	intercept	-1083.80	646.04	-1.68	0.12	0.12	0.12
	year	0.54	0.32	1.68	0.12		
pygmy	**intercept**	**-520.41**	**193.85**	**-2.69**	**0.02**	**0.31**	**0.02**
	**year**	**0.26**	**0.10**	**2.70**	**0.02**		

Table S3. Complete results of abundance ~ temperature + salinity + oxygen + chlorophyll *a* + year logistic regression models for a. targeted and b. untargeted species. Coefficients that are significant at p < 0.10 level are in bold.

S3a

species	coefficient	estimate	se	z	p
copper	intercept	-223.12	143.87	-1.55	0.12
	temperature	**-0.55**	**0.33**	**-1.64**	**0.10**
	salinity	0.88	2.05	0.43	0.67
	oxygen	0.24	0.66	0.37	0.71
	chlorophyl *a*	**0.49**	**0.21**	**2.32**	**0.02**
	year	**0.10**	**0.05**	**1.89**	**0.06**
widow	intercept	-12.80	114.41	-0.11	0.91
	temperature	**-0.90**	**0.31**	**-2.88**	**0.004**
	salinity	-1.32	1.49	-0.89	0.38
	oxygen	0.51	0.59	0.87	0.38
	chlorophyl a	0.00	0.25	-0.01	0.99
	year	0.03	0.04	0.70	0.48
chilipepper	intercept	-123.31	121.12	-1.02	0.31
	temperature	-0.20	0.32	-0.65	0.52
	salinity	**-3.21**	**1.32**	**-2.43**	**0.02**
	oxygen	-0.90	0.63	-1.42	0.15
	chlorophyl *a*	-0.02	0.33	-0.06	0.95
	year	**0.12**	**0.06**	**2.09**	**0.04**
blue	intercept	-141.40	96.39	-1.47	0.14
	temperature	**-1.08**	**0.26**	**-4.13**	**0.00004**
	salinity	-0.38	1.33	-0.29	0.77
	oxygen	**1.02**	**0.48**	**2.13**	**0.03**
	chlorophyl *a*	0.25	0.18	1.42	0.16
	year	**0.08**	**0.04**	**2.18**	**0.03**
speckled	intercept	**-215.70**	**97.28**	**-2.22**	**0.03**
	temperature	**-0.44**	**0.24**	**-1.87**	**0.06**
	salinity	-0.64	1.30	-0.49	0.62
	oxygen	0.00	0.48	0.01	0.99
	chlorophyl *a*	0.09	0.21	0.46	0.65
	year	**0.12**	**0.04**	**3.07**	**0.002**
bocaccio	intercept	-62.04	76.13	-0.82	0.42
	temperature	**-0.74**	**0.20**	**-3.63**	**0.0003**
	salinity	-1.47	0.97	-1.51	0.13
	oxygen	0.31	0.40	0.79	0.43
	chlorophyl *a*	-0.04	0.18	-0.24	0.81
	year	**0.06**	**0.03**	**1.92**	**0.06**
bank	intercept	**-174.40**	**78.37**	**-2.23**	**0.03**
	temperature	-0.12	0.16	-0.77	0.44
	salinity	0.25	0.97	0.26	0.79
	oxygen	0.54	0.41	1.31	0.19
	chlorophyl *a*	-0.20	0.22	-0.92	0.36
	year	**0.08**	**0.03**	**2.60**	**0.01**
olive	intercept	-91.20	108.39	-0.84	0.40
	temperature	**-0.63**	**0.28**	**-2.21**	**0.03**
	salinity	-1.14	1.41	-0.81	0.42
	oxygen	0.24	0.55	0.44	0.66
	chlorophyl *a*	0.14	0.22	0.66	0.51
	year	0.07	0.04	1.53	0.13

S3b

species	coefficient	estimate	se	z	p
swordspine	**intercept**	**-215.97**	**98.79**	**-2.19**	**0.03**
	temperature	0.01	0.15	0.08	0.93
	**salinity**	**3.14**	**1.32**	**2.38**	**0.02**
	oxygen	0.35	0.48	0.74	0.46
	chlorophyl *a*	-0.41	0.29	-1.41	0.16
	year	0.05	0.03	1.53	0.13
square spot	**intercept**	**-137.86**	**64.02**	**-2.15**	**0.03**
	temperature	-0.16	0.13	-1.27	0.20
	salinity	0.04	0.80	0.05	0.96
	oxygen	-0.51	0.33	-1.56	0.12
	**chlorophyl *a***	**0.29**	**0.16**	**1.86**	**0.06**
	**year**	**0.07**	**0.02**	**2.81**	**0.005**
shortbelly	intercept	-94.37	67.90	-1.39	0.16
	temperature	0.01	0.15	0.07	0.94
	salinity	-1.26	0.82	-1.53	0.13
	**oxygen**	**-1.27**	**0.36**	**-3.50**	**0.0005**
	**chlorophyl *a***	**0.49**	**0.17**	**2.95**	**0.003**
	**year**	**0.07**	**0.03**	**2.56**	**0.01**
whitespeckled	**intercept**	**-225.87**	**114.92**	**-1.97**	**0.05**
	**temperature**	**-0.68**	**0.28**	**-2.41**	**0.02**
	salinity	-0.28	1.56	-0.18	0.86
	oxygen	0.92	0.62	1.48	0.14
	**chlorophyl *a***	**-1.45**	**0.62**	**-2.35**	**0.02**
	**year**	**0.12**	**0.05**	**2.59**	**0.01**
stripetail	intercept	-38.60	77.56	-0.50	0.62
	temperature	-0.10	0.17	-0.62	0.54
	salinity	-0.99	0.94	-1.05	0.29
	oxygen	-0.63	0.40	-1.56	0.12
	chlorophyl *a*	0.28	0.18	1.59	0.11
	year	0.04	0.03	1.19	0.24
halfbanded	**intercept**	**-428.14**	**131.57**	**-3.25**	**0.001**
	temperature	0.22	0.29	0.73	0.46
	salinity	-2.24	1.45	-1.55	0.12
	**oxygen**	**-1.31**	**0.60**	**-2.20**	**0.03**
	**chlorophyl *a***	**0.61**	**0.23**	**2.69**	**0.01**
	**year**	**0.25**	**0.06**	**4.03**	**0.0001**
pygmy	intercept	-24.89	75.16	-0.33	0.74
	**temperature**	**-0.74**	**0.19**	**-3.82**	**0.0001**
	salinity	-0.87	0.99	-0.88	0.38
	oxygen	0.10	0.38	0.26	0.79
	chlorophyl *a*	-0.03	0.17	-0.16	0.87
	year	0.03	0.03	1.06	0.29

Table S4. Complete ANCOVA results for mean abundance ~ year + CCA + year*CCA models for a. targeted and b. untargeted species. Coefficients with p < 0.10 are in bold.

S4a.

species	coefficient	Type III SS	DF	F	p
speckled	intercept	0.20	1.00	0.22	0.64
	year	0.21	1.00	0.22	0.64
	**cca**	**27.38**	**1.00**	**29.23**	**0.00001**
	**year*cca**	**27.61**	**1.00**	**29.48**	**0.00001**
bocaccio	intercept	20.29	1.00	1.10	0.30
	year	20.42	1.00	1.11	0.30
	cca	3.21	1.00	0.17	0.68
	year*cca	3.13	1.00	0.17	0.68
bank	intercept	0.12	1.00	0.03	0.87
	year	0.11	1.00	0.03	0.87
	**cca**	**24.21**	**1.00**	**5.73**	**0.024**
	**year*cca**	**24.40**	**1.00**	**5.77**	**0.024**
olive	intercept	0.00	1.00	0.00	1.00
	year	0.00	1.00	0.00	1.00
	**cca**	**10.48**	**1.00**	**3.58**	**0.070**
	**year*cca**	**10.56**	**1.00**	**3.60**	**0.069**

S4b.

species	coefficient	Type III SS	DF	F	p
swordspine	intercept	0.86	1.00	0.27	0.61
	year	0.88	1.00	0.27	0.61
	cca	2.45	1.00	0.77	0.39
	year*cca	2.43	1.00	0.76	0.39
square spot	intercept	81.00	1.00	0.09	0.77
	year	81.60	1.00	0.09	0.77
	cca	105.60	1.00	0.12	0.74
	year*cca	111.80	1.00	0.12	0.73
shortbelly	intercept	1028.20	1.00	1.92	0.18
	year	1033.90	1.00	1.93	0.18
	cca	284.60	1.00	0.53	0.47
	year*cca	283.50	1.00	0.53	0.47
whitespeckled	intercept	2.59	1.00	0.35	0.56
	year	2.62	1.00	0.35	0.56
	cca	15.39	1.00	2.06	0.16
	year*cca	15.46	1.00	2.07	0.16
stripetail	intercept	2.97	1.00	0.26	0.62
	year	3.06	1.00	0.26	0.61
	cca	0.00	1.00	0.00	1.00
	year*cca	0.00	1.00	0.00	1.00
halfbanded	**intercept**	**6.20**	**1.00**	**2.86**	**0.10**
	**year**	**6.24**	**1.00**	**2.88**	**0.10**
	cca	1.14	1.00	0.53	0.47
	year*cca	1.15	1.00	0.53	0.47
pygmy	intercept	12.66	1.00	1.30	0.27
	year	12.75	1.00	1.31	0.26
	cca	6.02	1.00	0.62	0.44
	year*cca	5.87	1.00	0.60	0.45
