# Supplementary material for: Larval abundances of rockfishes that were historically targeted by fishing increased over 16 years in association with a large marine protected area
Source: R Soc Open Sci. 2017 Sep 20;4(9):170639. doi: 10.1098/rsos.170639 (PMC5627106; doi:10.1098/rsos.170639)
Supplement: Supplemental Figures for Thompson et al. 2017 [file rsos170639supp3.pdf]

Supplementary figure legends

Thompson et al. 2017 Production of rockfish historically targeted by fishing has increased in a large marine protected area over 16 years. Proc B

Figure S1. Histograms showing frequency of occurrence of abundances for 4 species with an extreme outlier station. This station, which was sampled in 2004, was removed from the ANOVA and ANCOVA analyses.

Figure S2. Two-way dendrogram grouping stations by habitat similarity. Each variable was standardized to mean unit variance and are thus on the same scale. Station labels depict the CalCOFI line (e.g., 93.3) and station (e.g., 45). Stations highlighted in yellow are within the CCAs. Stations highlighted in azure had similar environmental conditions to the CCA stations and were used in the ANCOVA to determine if rates of change in abundance varied inside versus outside of the CCAs.

Figure S3. Mean density of blue rockfish larvae at each station averaged over the 16-year duration of the study. White points depict locations where at least 1 larva was found, and sizes are scaled based on mean abundance (range: 0.38 to 26.5). Yellow points are locations where blue rockfish larvae were never found.

Figure S4. Mean abundance of untargeted larvae within (red) and outside (blue) of the CCAs. Mean abundance ~ year + CCA + CCA*year for each species was modeled with an ANCOVA, and there were no significant interactions between CCA and year for any species.

figure S1

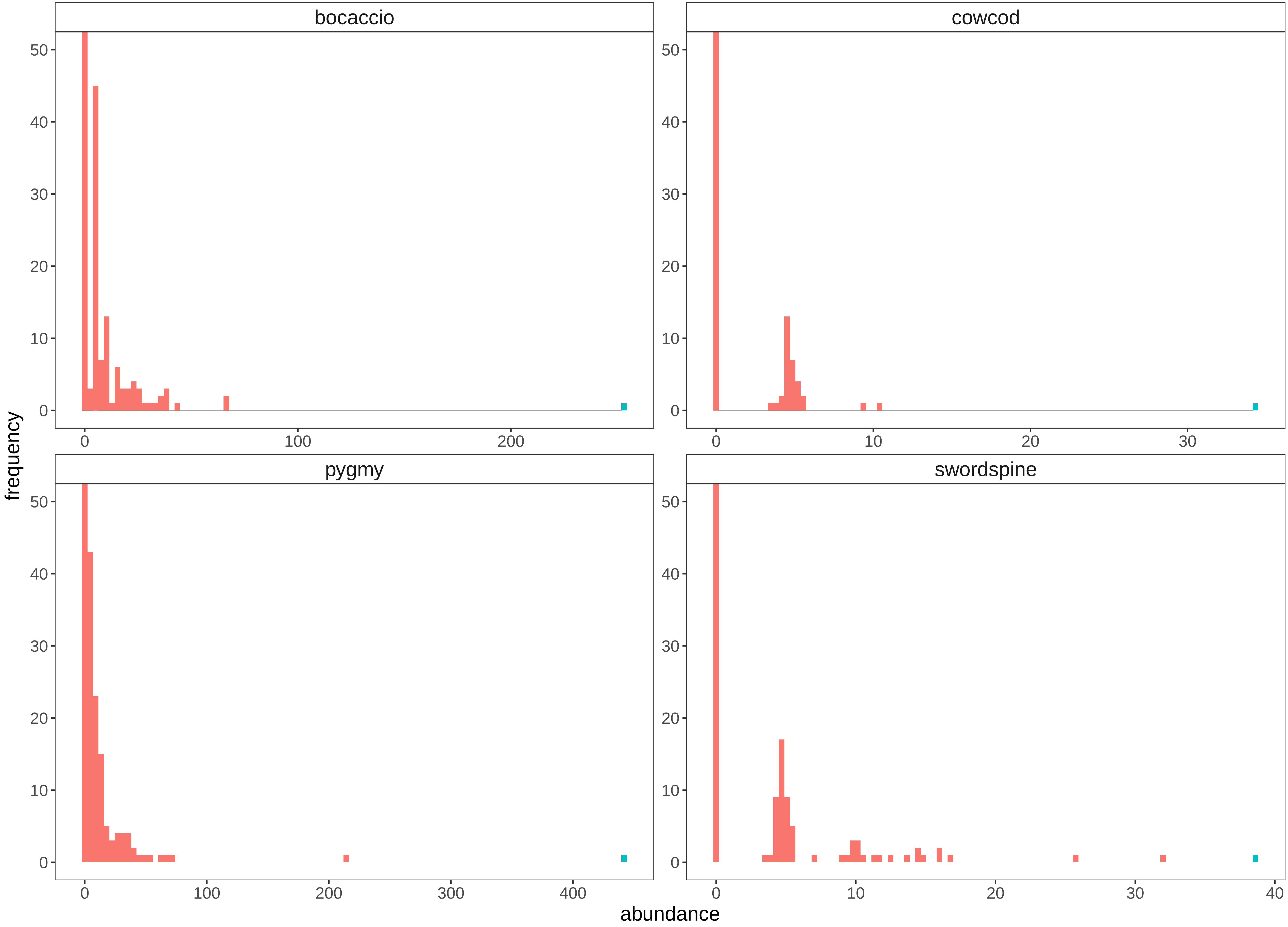

bocaccio
cowcod
pygmy
swordspine
frequency
abundance
50
40
30
20
10
0
0
100
200
300
400
10
20
30
40

figure S2

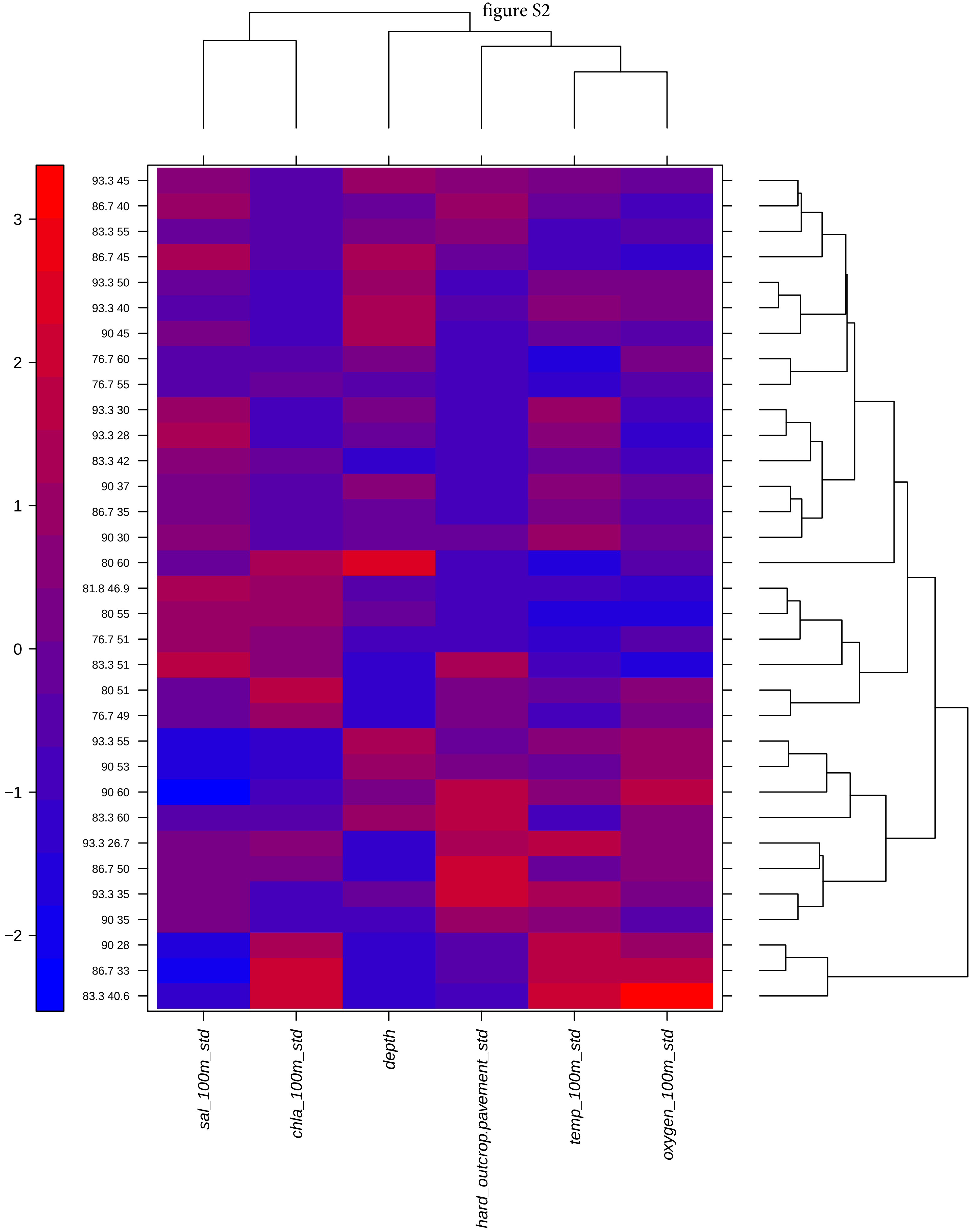
3
2
1
0
−1
−2
93.3 45
86.7 40
83.3 55
86.7 45
93.3 50
93.3 40
90 45
76.7 60
76.7 55
93.3 30
93.3 28
83.3 42
90 37
86.7 35
90 30
80 60
81.8 46.9
80 55
76.7 51
83.3 51
80 51
76.7 49
93.3 55
90 53
90 60
83.3 60
93.3 26.7
86.7 50
93.3 35
90 35
90 28
86.7 33
83.3 40.6
sal_100m_std
chla_100m_std
depth
hard_outcrop.pavement_std
temp_100m_std
oxygen_100m_std

figure S3

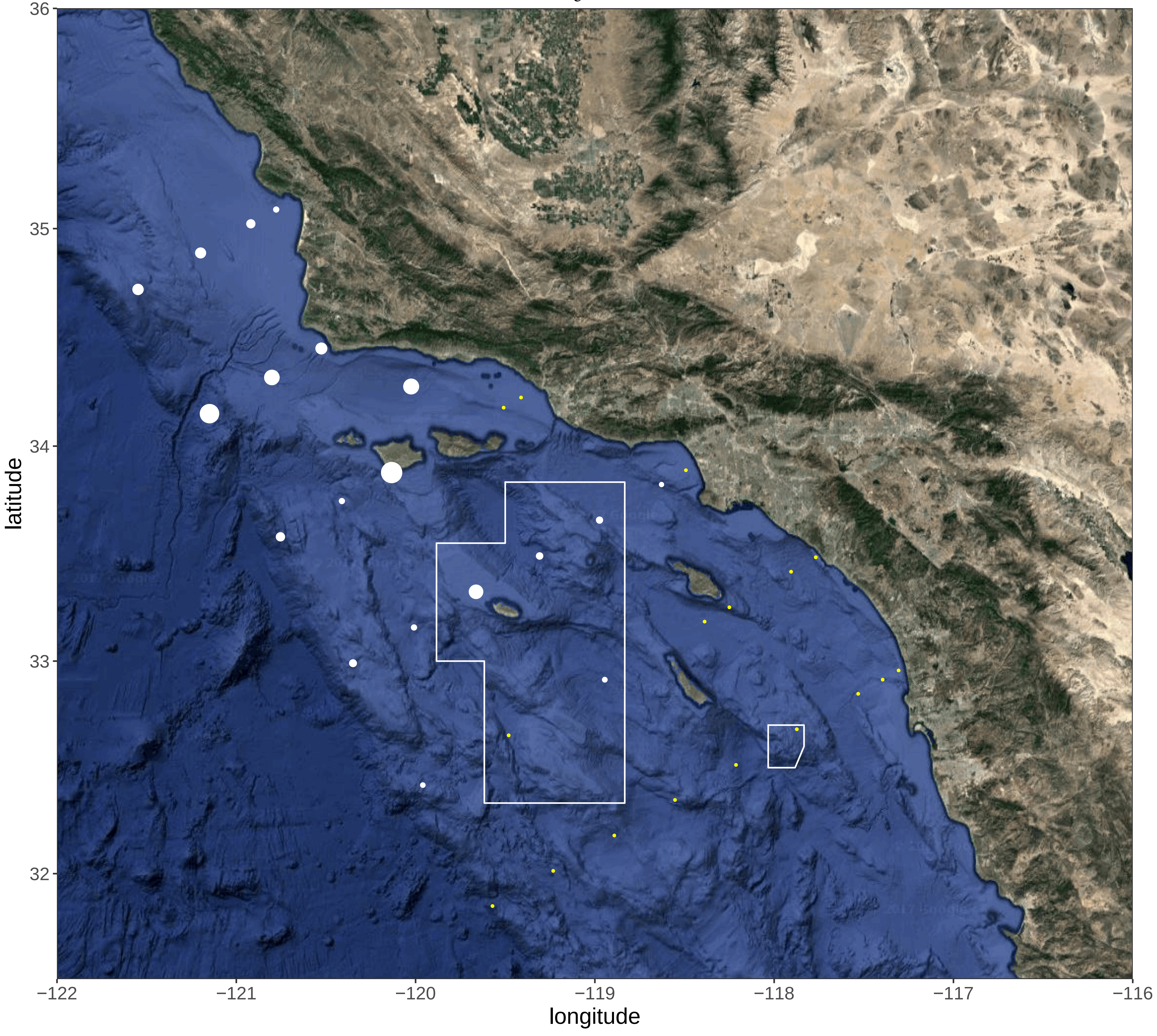

figure S4

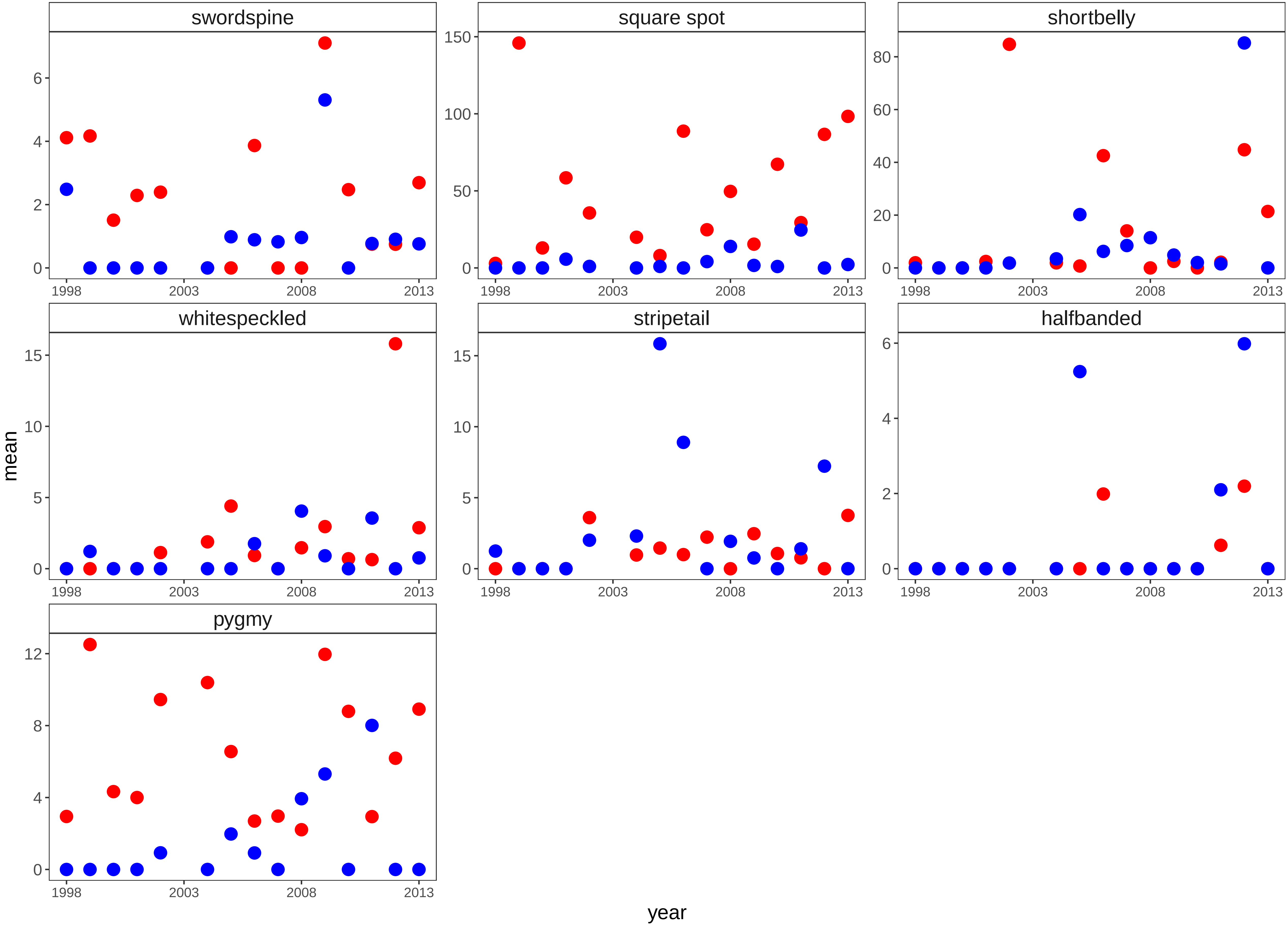

swordspine
square spot
shortbelly
whitespeckled
stripetail
halfbanded
pygmy
mean
year
1998
2003
2008
2013
