# Supplementary material for: Larval abundances of rockfishes that were historically targeted by fishing increased over 16 years in association with a large marine protected area
Source: R Soc Open Sci. 2017 Sep 20;4(9):170639. doi: 10.1098/rsos.170639 (PMC5627106; doi:10.1098/rsos.170639)
Supplement: Supplemental Appendices for Thompson et al. 2017 [file rsos170639supp4.pdf]

Supplementary Appendices

Thompson et al. 2017 Production of rockfish historically targeted by fishing has increased in a large marine protected area over 16 years. Proc B

Appendix S1. Methods to genetically-identify rockfish larvae to species

Genomic DNA from each larva was extracted using a chelex-based boiling protocol [1]. Tissue was typically taken from the eye, but was taken from the posterior region of the larva if eyes were missing. Individuals were retained in separate vials for future, planned otolith analyses. Polymerase chain reaction (PCR) using the primers GLURF2-5' and CB3RF2-5' was used to amplify 625 base pairs of the mitochondrial cytochrome *b* gene [2]. PCRs were conducted in 10 µL volumes with buffer (67 mM Tris-HCl pH 8.8, 16.6 mM (NH4)2SO4, 10 mM β-mercaptoethanol, 2 mM MgCl2), 800 µM dNTP, 0.4 µM of each primer, 0.5 mg/ml bovine serum albumin, 0.5 units *Taq* DNA polymerase (New England Biolabs), and 1 µL of chelex supernatant containing DNA template. The thermal profiles of the PCRs were: denature at 92°C for 2:30; followed by 40 cycles of 94°C for 0:30, 55°C for 1:30, 70°C for 1:30; then a final extension of 72°C for 3:00. Negative controls with no template DNA were run for each PCR to ensure that there was no contamination (there were no signs of contamination). ExoSap-IT (Affymetrix) was used to enzymatically clean PCR products according to manufacturer's protocol. The cleaned products were then sequenced in one direction using the internal primer CBINR3-5' (ATGAGAARTAGGGGTGGAAGCT) and BigDye v3.1 Dye Terminator chemistry following manufacturer's protocols, and analyzed with an ABI3730 Genetic Analyzer (Life Technologies). Sequences were edited and aligned using Sequencher v4.9 (GeneCodes),

aligned with templates from reference adult rockfishes [3] and identified by creating Neighbor Joining phylogenetic trees with MEGA v6.06 [4].

Appendix S2. R packages used to make maps, graphs, and conduct statistical analyses

All plots and analyses were conducted using program R v3.3.2 [5]. We are deeply appreciative to the authors of the R packages that we utilized. Data manipulation was accomplished using the packages reshape2 [6]and plyr [7]. We tested for spatial autocorrelation using rgdal [8] and ncf [9]. Delta means were calculated using fishmethods [10], and type III ANOVA was run using the car [11] pacakge. The package vegan [12] was used to transform abundance data to presence/absence for logistic regression while outliers [13] detected the major outlier station in 2004. All plots were made with ggplot2 [14] with the exception of the 2-way dendrogram that was created using lattice [15] and latticeExtra [16] and the map which was made using ggmap [17].

1. Hyde J.R., Lynn E., Humphreys R., Musyl M., West A.P., Vetter R. 2005 Shipboard identification of fish eggs and larvae by multiplex PCR, and description of fertilized eggs of blue marlin, shortbill spearfish, and wahoo. *Marine Ecology Progress Series* **286**, 269-277.
2. Hyde J.R., Kimbrell C.A., Budrick J.E., Lynn E.A., Vetter R.D. 2008 Cryptic speciation in the vermilion rockfish (Sebastes miniatus) and the role of bathymetry in the speciation process. *Molecular Ecology* **17**, 1122-1136.
3. Hyde J.R., Vetter R.D. 2007 The origin, evolution, and diversification of rockfishes of the genus *Sebastes* (Cuvier). *Molecular Phylogenetics and Evolution* **44**, 790-811.
4. Tamura K., Stecher G., Peterson D., Filipski A., Kumar S. 2013 MEGA6: Molecular Evolutionary Genetics Analysis version 6.0. *Molecular Biology and Evolution* **30**, 2725-2729.
5. R_Core_Team. 2016 R: A language and environment for statistical computing. (ed. R_Foundation_for_Statistical_Computing). Vienna, Austria.
6. Wickham H. 2007 Reshaping data with the reshape package. *Journal of Statistical Software* **21**, 1-20.
7. Wickham H. 2011 The split-apply-combine strategy for data analysis. *Journal of Statistical Software* **40**, 1-29.
8. Bivand R.S., Keitt T., Rowlingson B. 2016 rgdal: Bindings for the geospatial data abstraction. (pp. R package version 1.2-5.

9. Bjornstad O.N. 2016 ncf: spatial nonparametric covariance functions. (pp. R package version 1.1-7.
10. Nelson G. 2016 fishmethods: Fishery science methods and models in R. (pp. R package version 1.2-5.
11. Fox J., Weisberg S. 2011 *An R companion to applied regression, second edition*. Thousand Oaks, CA, Sage.
12. Oksanen J., Blanchet F.G., Friendly M., Kindt R., Legendre P., McGlinn D., Minchin P.R., O'Hara R.B., Simpson G.L., Solymos P., et al. 2017 vegan: Community Ecology Package. (2.4-2 ed.
13. Komsta L. 2011 outliers: Tests for outliers. R package version 0.14. (
14. Wickham H. 2009 *ggplot2: elegant graphics for data analysis*, Springer New York.
15. Sarkar D. 2008 *Lattice: Multivariate Data Visualization with R*. New York, Springer.
16. Sarkar D., Andrews F. 2016 latticeExtra: Extra graphical utilities based on lattice. R package version 0.6-28. (http://CRAN.R-project.org/package=latticeExtra.
17. Kahle D., Wickham H. 2013 ggmap: Spatial visualization with ggplot2. *The R Journal* **5**(1), 144-161.
